# Lesion Analysis in PERCIST 1.0: Clinical Ease versus Research Requisite—Where Does the Balance Exist?

**DOI:** 10.1055/s-0042-1750406

**Published:** 2023-04-28

**Authors:** Amit Bhoil

**Affiliations:** 1 Department of Nuclear Medicine, Mahajan Imaging and Labs, New Delhi, India

**Keywords:** NSCLC, PERCIST 1.0, SUV, TLG, MTB, tumor burden, radiomics, lesion analysis

## Abstract

**Background** Semiqualitative parameter SUVmax has been the most frequently used semiquantitative positron emission tomography (PET) parameter for response evaluation, but only metabolic activity of a single (most metabolic) lesion is predicted. Newer response parameters such as tumor lesion glycolysis (TLG) incorporating lesions' metabolic volume or whole-body metabolic tumor burden (MTBwb) are being explored for response evaluation. Evaluation and comparison of response with different semiquantitative PET parameters such as SUVmax and TLG in most metabolic lesion, multiple lesions (max of five), and MTBwb in advanced non-small cell lung cancer (NSCLC) patients were made. The different PET parameters were analyzed for response evaluation, overall survival (OS), and progression-free survival (PFS).

**Methods** ^18^ F-FDG-PET/CT (18-fluorine-fluorodeoxyglucose positron emission tomography/computed tomography) imaging was performed in 23 patients (M = 14, F = 9, mean age = 57.6 years) with stage IIIB–IV advanced NSCLC before initiation of therapy with oral estimated glomerular filtration rate-tyrosine kinase inhibitor for early and late response evaluation. The quantitative PET parameters such as SUVmax and TLG were measured in single (most metabolic) lesion, multiple lesions, and MTBwb. The parameters SUVmax, TLG, and MTBwb were compared for early and late response evaluation and analyzed for OS and PFS

**Results** No significant difference in change in response evaluation was seen in patients evaluated with most metabolic lesion, multiple lesions, or MTBwb. Difference in early (DC 22, NDC 1) and late (DC 20, NDC 3) response evaluation was seen that remained unchanged when lesions were measured in terms of number of lesions or the MTBwb. The early imaging was seen to be statistically significant to the OS compared with late imaging.

**Conclusions** Single (most metabolic) lesion shows similar disease response and OS to multiple lesions and MTBwb. Response evaluation by late imaging offered no significant advantage compared with early imaging. Thus, early response evaluation with SUVmax parameter offers a good balance between clinical ease and research requisition.

## Key Message

Early response evaluation with SUVmax measurement of single lesion offers a good balance between clinical ease and research requisition compared with whole-body tumor metabolic burden.

## Introduction

Non-small cell lung cancer (NSCLC) treatment remains a challenge with approximately 75% patients presenting with advanced disease. 1 Tumor shrinkage with improved survival outcomes with newer cytostatic drugs is seen in only 10 to 15% patients, with the clinical imaging playing a significant role in patient management. Evolving newer therapies brought the need for standardization of response criteria for assessment of cancer treatment, with multiple response criteria, such as World Health Organization (WHO), RECIST, RECIST1.1, EASL criteria, Choi criteria, and PERCIST 1.0 criteria. 2

^18^ F-FDG PET/CT (18-fluorine-fluorodeoxyglucose positron emission tomography/computed tomography) imaging has been recommended by various guidelines for tumor management. Qualitative visual criteria, Deauville 5-point visual scale criteria, and a 4-point scale in colorectal cancer qualitative assessment were progressive steps in response assessment. 2 3 4

The current PET workstations routinely use the semiquantitative variables such as SUVmax, SUVmean, and SUVpeak as quantitative treatment response parameters in clinical assessment, with the advantage of being resistant to partial volume effect in small-sized tumors. However, these semiquantitative variables are highly dependent on the statistical quality of images and maximal pixel size, often neglecting the lesion's dimension and total composition of the affected nodal and extranodal sites. 5 The total lesion volume and its metabolic activity, known as the total lesion glycolysis, effective glycolytic volume, or total glycolytic volume parameters, have been some of the other important semiqualitative parameters toward studying the tumor behavior. 6 7 8 Different primary tumor sites or metastatic sites may present with different responses as seen in some of the tumors such as renal carcinoma, with inclusion of the primary disease site seen to impact response and time to progression. 9 Thus, theoretically assessment of the tumor burden encompassing multiple sites of target lesion is advocated for the disease measurement and reproducibility. 10

Advanced methods of assessment of tumor burden such as metabolic tumor volume (MTV), tumor lesion glycolysis (TLG), and whole-body metabolic tumor burden (MTBwb) have also been considered for assessment of response and prognostication. In some of the malignancies such as lung, esophageal carcinoma, and mesothelioma, MTV is seen to be a better and independent prognostic factor and predictor of survival than SUVmax. 11 Assessment of TLG being the combination of MTV and SUVmean indicates the degree of ^18^ F-FDG uptake and the size of the metabolically active tumor appearing as an ideal metabolic parameter to reflect total tumor burden of the lesion. The MTV and whole-body TLG (TLGwb) or the whole-body MTV (MTVwb) are other important indexes of the overall malignant process in the body. The MTBwb has been shown to have a prognostic value for NSCLC patients, beyond TNM stage and other factors such as age, performance status, and tumor histology being relatively immune to the effect of interobserver variability. 12 13 14 15

The PET/CT-based volumetric prognostic index (PVP index) combining the MTVwb and TNM stage prognostication has been proposed by some researchers 12 as a practical means for clinicians to combine the prognostic value of MTVwb and TNM stage, offering a better prognostic accuracy for overall survival (OS) of NSCLC patients than the current TNM staging system or metabolic tumor burden alone. The metabolic response evaluation with lesion analysis with parameters such as SUVmax, TLG, MTV, MTVwb, and MTBwb has its advantages and disadvantages but no effort to our knowledge has been made to analyze different numbers of lesion parameters and directly compare the results between all these methodologies for response evaluation and role in OS to strike a fine balance between clinical needs and research requisite.

With these objective, the retrospective study data were analyzed with the aim to explore the comparative role of single (most metabolic) tumor lesion, multiple metabolic lesions (max of five), and MTBwb for the assessment of the response at early (21 days) and at late (42 day) time intervals by PERCIST 1.0 criteria.

## Subjects and Methods

### Patients

Histologically proven adenocarcinoma NSCLC patients (stages IIIB and IV) for initiation of estimated glomerular filtration rate-tyrosine kinase inhibitor (EGFR-TKI) as the first, second/third line of treatment were included in the study. 16 17 All patients underwent baseline investigations that included complete physical examination, ECOG status, biochemical assessment and histopathological examination, and baseline ^18^ F-FDG PET/CT imaging prior to starting oral EGFR-TKI ( Table 1 ). Patients who had received prior treatment with oral EGFR-TKI or were allergic and/or intolerant to these drugs were excluded from the study. Follow-up scans were done at an early and a late time period and the PFS and OS of disease control (DC) and no DC (NDC) taken as the end point of the study. The work was performed involving human participants as per the clinical treatment guidelines in accordance with ethical standards of national research committee and complied with 1964 Declaration of Helsinki and its later amendments. All patients gave informed consent prior to treatment and management. No additional ethics approval was therefore required.

**Table 1:** Patient characteristics

Characteristic	Total patients ( *n* = 23)
Age (y) a	55 (28–86)
Follow-up period (d) a	399 (5–1,761)
Male	14
Female	6
Histology	
Adenocarcinoma	23
Tyrosine kinase inhibitor	
Gefitinib (250 mg)	14
Erlotinib (150 mg)	9
Indication for treatment	
First line	7
Second and third lines	16

a Data are median (range).

### Treatment

Patients received an oral dose of either gefitinib (250 mg) or erlotinib (150 mg) daily as per the established protocol. 18 If disease progressed, treatment was discontinued; in case of drug toxicity the dose was reduced to every alternate day and was stopped in case of severe toxicity, like intolerable side effects. Treatment was resumed only if the patient recovered from drug toxicity in less than 2 weeks.

### PET/CT Acquisition Protocol and Image Analysis

Baseline ^18^ F-FDG PET imaging was done prior to starting the oral EGFR TKI therapy, after 21 days and 42 days of treatment with oral EGFR TKI. Imaging by PET/CT was performed in three-dimensional (3D) mode using a dedicated PET/CT scanner (Discovery STE-16, GE, Milwaukee, United States) at a median uptake time 64 minutes (range: 61–101 minutes) following an intravenous injection of ^18^ F-FDG with a mean administered activity of 374.0 MBq (range: 261.59–475.82 minutes). All patients were kept fasting for at least 6 hours before the 18F-FDG injection and blood glucose levels were always kept within 200 mg/dL.

Whole-body scans were acquired in overlapped bed positions from skull to mid-thigh and 1 to 2 minute acquisition was performed for each bed position. CT was performed after injection of contrast media using a tube current of 115 mAs and a voltage of 130 kVp. After transmission scan, 3D PET acquisitions were done for 1 to 2 minutes per bed position. Image reconstruction was done using iterative reconstruction algorithm. The transaxial, coronal, and sagittal images were obtained after reconstruction. The study protocol, image acquisition, and image reconstruction remained identical for both baseline and progressive scans.

Two experienced nuclear medicine physicians determined the tumor primary site, nodal, and/or a distant metastasis. These quantitative uptake values were calculated in the form of SUVmax, and TLG, MTV, MTVwb, TLGwb, and MTBwb using software as AW VolumeShare 5 (AW4.6) and RadiAnt DICOM Viewer 4.2.1 (Medixant, Poznan, Poland) ( https://www.radiantviewer.com ).

### Response Assessment Using PET Imaging

The output results included the SUVmax, SUVmean, MTV, and TLG of individual tumor lesions and multiple combined tumor lesions. The lesions with the highest SUVmax were identified on the baseline PET images and compared with the lesions with the highest SUVmax on the follow-up PET images for evaluation of response. The percentage changes of these parameters and residual values from a single PET study early and late imaging during treatment were used for treatment response prediction and classifying responses as proposed by the PERCIST 1.0 response criteria. 2 All patients who showed complete metabolic response (CMR), partial metabolic response (PMR), or stable metabolic disease (SMD) were categorized as having DC and patients with progressive metabolic disease (PMD) were categorized under NDC ( Tables 2 and 3 ).

**Table 2:** PERCIST disease classification with SUVmax parameter response evaluation with single (most metabolic) tumor lesion, multiple lesions (max. five) and MTBwb for response evaluation

Lesions SUV _max_ response	SUV _max_ of single (most metabolic) lesion		Summed SUV _max_ of multiple lesions (maximum of 5 lesions)		Whole body metabolic tumor burden (MTBwb)	
Response	Early imaging	Late imaging	Early imaging	Late imaging	Early imaging	Late imaging
SMD	13	12	13	12	13	12
PMR	9	7	9	8	9	8
CMR	0	1	0	0	0	0
PMD	1	3	1	3	1	3
DC	22	20	22	20	22	20
NDC	1	3	1	3	1	3

Abbreviations: CMR, complete metabolic response; DC, disease control; NDC, no disease control; PMD, progressive metabolic disease; PMR, partial metabolic response; SMD, stable metabolic disease.

**Table 3:** PERCIST disease classification with TLG parameter response evaluation with single (most metabolic) tumor lesion, multiple lesions (max. five) and MTBwb for response evaluation

Lesion TLG response	TLG of single (most metabolic) lesion		Summed TLG of multiple lesions (maximum of 5 lesions)		Whole body metabolic tumor burden (MTBwb)	
Response	Early	Late	Early	Late	Early	Late
SMD	13	11	14	13	14	13
PMR	6	7	8	8	8	8
CMR	0	1	0	1	0	1
PMD	4	4	1	1	1	1
DC	19	19	22	22	22	22
NDC	4	4	1	1	1	1

Abbreviations: CMR, complete metabolic response; DC, disease control; NDC, no disease control; PMD, progressive metabolic disease; PMR, partial metabolic response; SMD, stable metabolic disease.

**Fig. 1:**
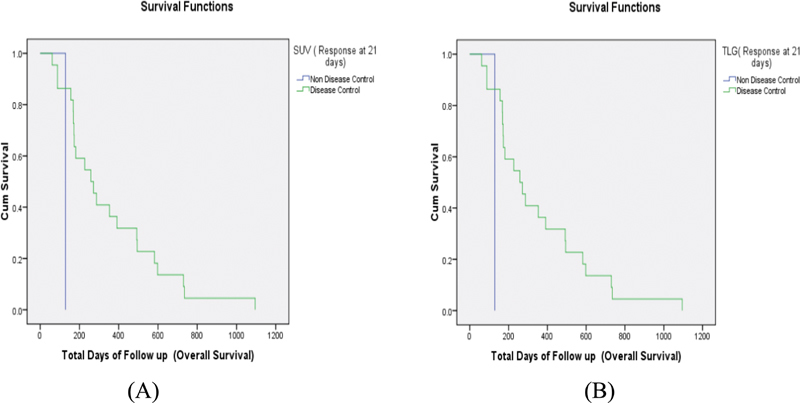
Kaplan Meier curves in OS in Disease control (DC) vs. No Disease Control (NDC). (A) SUVmax response evaluation with single lesion at early response ( *p* = 0.049); (B) Summed TLG of multiple lesions and MTBwb with early and late response ( *p* = 0.049)

The PFS and OS were estimated for DC and NDC groups ( Tables 4 and 5 ) and the significance of SUVmax for both ^18^ F-FDG and TLG of MTVwb for the prediction of OS was estimated using Kaplan–Meier analysis ( Fig. 1 ). Logistic regression analysis was applied to see if PFS and OS correlated with various parameters.

**Table 4:** PFS and OS with response evaluated with SUVmax with disease classified as SMD, PMR, CMR (DC), and PMD (NDC) in early and late imaging

SUV _max_	Single (most metabolic) lesion				Summed SUV _max_ of multiple lesions (maximum of 5 lesions)				Whole body metabolic tumor burden (MTBwb)			
	Early imaging		Late imaging		Early imaging		Late imaging		Early imaging		Late imaging	
	DC (22)	NDC (1)	DC (20)	NDC (3)	DC (22)	NDC (1)	DC (20)	NDC (3)	DC (22)	NDC (1)	DC (20)	NDC (3)
Median PFS (d)	172 (101–243)	128	169 (151–187)	172 (102–242)	172 (101–243)	128	169 (151–187)	172 (102–242)	172 (101–243)	128	169 (151–187)	172 (102–242)
*p* -Value	0.183		0.461		0.183		0.461		0.183		0.461	
Median OS (d)	258 (136–380)	129	271 (140–402)	172 (103–241)	258 (136–380)	129	271 (140–402)	172 (103–241)	258 (136–380)	129	271 (140–402)	172 (103–241)
*p* -Value	0.049		0.115		0.049		0.115		0.049		0.115	

Abbreviations: DC, disease control; NDC, no disease control; OS, overall survival; PFS, progression-free survival.

**Table 5:** PFS and OS with response evaluated with TLG with disease classified as SMD, PMR, CMR (DC), and PMD (NDC) in early and late imaging

TLG	Single (most metabolic) lesion				Summed TLG of multiple lesions (maximum of 5 lesions)				Whole body metabolic tumor burden (MTBwb)			
	Early imaging		Late imaging		Early imaging		Late imaging		Early imaging		Late imaging	
	DC (19)	NDC (4)	DC (19)	NDC (4)	DC (22)	NDC (1)	DC (22)	NDC (1)	DC (22)	NDC (1)	DC (22)	NDC (1)
Median PFS (d)	172 (149–195)	165 (0–541)	172 (149–195)	165 (0–541)	172 (101–243)	128	172 (101–243)	128	172 (101–243)	128	172 (101–243)	128
*p* -Value	0.172		0.172		0.183		0.183		0.183		0.183	
Median OS (d)	227 (106–348)	353 (0–812)	227 (106–348)	353 (0–812)	258 (136–380)	129	258 (136–380)	129 (-)	258 (136–380)	129	258 (136–380)	129
*p* -Value	0.496		0.496		0.049		0.049		0.049		0.049	

Abbreviations: DC, disease control; NDC, no disease control; OS, overall survival; PFS, progression-free survival.

Patients' response was assessed at 3-month interval in view of the clinical status, and anatomical imaging (radiography, CT, or MRI) with RECIST 1.0. 2 The time to progression was calculated from initiation of EGFR-TKI to the first evidence of any disease progression.

## Statistical Analysis

Statistical analysis was performed using Statistical Package for Social Sciences software (SPSS Inc., Chicago, Illinois, United States, version 15.0). *p* -Value of less than 0.05 was considered as statistically significant. All quantitative variables were expressed as median, mean, and range, and standard deviation (SD) was also calculated. Median OS and PFS were estimated by Kaplan–Meier analysis. The time to progression and death served as endpoints. The PFS and OS were compared by the log-rank test.

## Results

### Patient Characteristics

Forty patients were enrolled in the study. All patients underwent histopathology, baseline CT, and ^18^ F FDG-PET/CT. Twenty-three patients underwent all three response assessment studies before treatment initiation and at early and late time intervals, 9 patients underwent two studies before treatment initiation and at 21 days, and 8 patients underwent single study before treatment initiation. Thus 23 patients (14 males and 9 females) with stage IIIb or higher disease and a mean age of 57.6 years (range: 28–86 years) of adenocarcinoma were included in the final analysis. EGFR mutation analysis was performed in all patients with samples suitable for molecular analysis.

A total of 120 lesions were analyzed on the baseline scans and on the corresponding early follow-up scans. One to five lesions per patients were analyzed (median; 4 lesions; range: 1–5 per patient). In the patient subgroup, with response evaluation using all three 18F FDG PET/CT imaging studies, 80 lesions were evaluated in 23 patients at baseline, early imaging and late imaging of PET/CT imaging follow-up studies (median: 3 lesions; range: 1–5 lesions).

Disease progression from PMR to PMD was seen in two patients during the late imaging with SUVmax analysis. However, no change in overall disease classification as DC and NDC was seen with SUVmax or TLG parameter when single (most metabolic) tumor lesion, multiple lesions (maximum of five), and MTBwb were examined for early or at late response ( Tables 2 and 3 ).

OS and PFS of response for most metabolic tumor lesion, multiple lesions (maximum of five), and MTBwb are shown below.

The OS was statistically significantly correlated to early imaging ( *p* = 0.049) compared with late imaging ( *p* = 0.115) when response was measured by SUVmax, but no statistical significance was noted with TLG ( *p* = 0.496). The PFS was not statistically significantly correlated to early ( *p* = 0.183) or the late imaging ( *p* = 0.461) when response was evaluated with either of the parameters: SUVmax (0.172), or TLG ( *p* = 0.183), or MTBwb ( *p* = 0.183) ( Tables 4 and 5 )

## Discussion

Tumor assessment with the newer cytostatic drugs has limitations with assessment using the RECIST criteria. 19 Metabolic response criteria incorporating the metabolism, volume quantification, and patient survival are considered to be more sensitive than the criteria such as the WHO, RECIST 1.0, and RECIST 1.1 criteria. 2 20

Early works on metabolic response evaluation were focused on setting of single tumor lesion analysis 21 and subsequently the multiple tumor foci were measured with an average of 2.2 lesions for response assessment. 22 23 24 The response assessment has evolved, to consider lesion volume metabolism measurements with the TLG and MTBwb to be better parameters for response evaluation and OS in some of the tumors. 25 26

The patients were retrospectively analyzed to compare the response with PERCIST 1.0 criteria with regard to examination of multiple lesions, as the single (most metabolic) lesion, multiple lesions (max of five lesions), and MTBwb. The single lesion and multiple metabolic lesions contemplated to predict the most metabolically aggressive biological behavior of the primary tumor. The changes in ΔSUV and ΔTLG of the most metabolic lesion were compared for the response. The metabolic change in MTBwb (ΔMTVwb) was also assessed for metabolic response evaluation.

Some researchers as such Benz et al 22 advocated summing the SUVmax, while concluding that summing of the lesion although shows a difference in the tumor lesion metabolic burden when measured with TLG but does not make any significant difference in SUVmax unless lesion transformation has occurred into a mutator phenotype. 2 With the hypothesis to understand any transformation into mutator phenotype in our patient group, we measured the single lesion and also summed SUVmax of the multiple lesions.

The patients classified as DC or NDC did not show any difference in response when most metabolic SUVmax was measured or the multiple lesions (max of five) summed SUVmax or MTBwb was measured for response assessment and in relation to the OS. Hence we could advocate the measurement of the ΔSUVmax between the tumor with the single lesion on the baseline study and follow-up studies to classify response evaluation. This methodology is not only more convenient but also free from any observer biases, which may develop while examining multiple lesions.

Metabolic parameters as MTV, TLG, and MTVwb have been correlated with the prognostication and response evaluation in different tumors as NSCLC, esophageal cancer, and lymphoma. 20 21 22 23 24 Different methods for quantification of TLG have been considered, such as the fixed thresholding of SUVmax with 3 SDs above normal liver, 2 the size-dependent threshold independent of tumor to background ratio for measurement of TLG, 25 or the lesion volume with the help of CT with the thresholding method. 26 The fixed thresholding method is preferred although it has a limitation that TLG of tumors with low glycolytic activity and tumor to background ratio cannot be calculated. The measurement of *K*_i_ index through dynamic study in some of the patients has seen to be an attractive parameter especially helpful when the SUV is low after treatment, 2 27 but is limited by time constrain, spatial location, and limited standard software availability mitigating its utility for routine clinical use. We believe visual assessment considered by Hicks qualitative PET criteria 2 may be helpful for determining the presence or absence of complete response, especially for small lesions after treatment.

The MTV has been seen as an independent poor prognostic factor in lung and head carcinoma, 28 29 suggesting that MTBwb as a parameter of MTV and TLG of whole body probably could be a better quantitative index of treatment response in some of the tumors than SUVmax. But no difference in response classification was seen in our patient groups when MTBwb was compared with the SUVmax of the most metabolic lesion. Difference in response classification was seen between patients classified as DC or NDC with ^18^ F-FDG PET/CT response when analysis was done with TLG between the single lesion (DC: 19, NDC: 4) and multiple tumor lesions (DC: 19, NDC: 4) or the MTBwb (DC: 22, NDC: 1), but none of the change in disease classification in MTBwb was statistically significant to OS. Thus, calculation of MTBwb showed no significant advantage in disease classification to OS while being tedious precluded its routine use in clinical setting.

Our analysis suggests that semiquantification of single lesion can be a preferable means of measurement without causing any meaningful difference in the response and clinical outcome. Our observation was shared with research study of Hussien et al, 30 where ΔSUVmax performed better than multiple parameters such as SUVmean, MTV, and TLG of PET measuring response assessment.

We consider that increasing the number of target lesions measured in an organ should reduce errors in metabolic volume quantification. 2 10 A necessary balance needs to be maintained between clinical reporting and carrying out a clinical trial in a busy department. All other factors being equal, measurement of fewer and least number of lesions may be a preferable option. Further, the single lesion measurement with technical advances could be used to reduce systemic errors rather than measuring multiple lesions, being more practical means of striking a balance in reporting.

Early response evaluation is considered to be more cost effective as seen by many researchers. 31 32 33 Some difference with regard to the response in disease classification with SUVmax at early imaging (DC: 22, NDC: 1) versus late imaging (DC: 20, NDC: 3) was seen in our study but early imaging was still statistically significant compared with the late imaging ( *p* = 0.049 vs. 0.115). No difference in the statistically significant OS ( *p* = 0.049) was seen during the response evaluation at the early time period or the late time period with TLG parameter.

Although our study found a good agreement with regard to tumor response rate with PERCIST 1.0 response assessment and OS when evaluated with single lesion, multiple lesions, or the MTBwb, yet we do consider that a similar observation in a larger group of patients shall be of considerable interest clinically especially when MTBwb has been observed to have low interobserver variability and prognostic measurement in patients. 14

In the current era of radiomics, inclusion of the textural analysis along with the PET/CT metabolic parameters for assessing any tumor heterogeneity in some cancers shall be an area of interest for evaluation of patient response and patient prognostication, extending the concept of radiogenomics. 34 The limited sample size and lack of textural analysis in patients presenting with progressive disease in follow-up response are some of the limiting factors of the study.

## Conclusion

Single most metabolic tumor lesion analysis shows similar response and OS to multiple lesions and MTBwb. Late imaging offered no significant advantage compared with early imaging in disease response evaluation. Thus, early response evaluation in single (most metabolic) lesion with SUVmax parameter could likely offer a balance between clinical ease and research requisition.
